# The Berlin Inventory of Gambling behavior – Screening (BIG-S): Validation using a clinical sample

**DOI:** 10.1186/s12888-017-1349-4

**Published:** 2017-05-18

**Authors:** Martin Wejbera, Kai W. Müller, Jan Becker, Manfred E. Beutel

**Affiliations:** Department of Psychosomatic Medicine and Psychotherapy, University Medical Center, Mainz, Germany

**Keywords:** Gambling disorder, Diagnostic tool, Screening, Validation, Usability, DSM V

## Abstract

**Background:**

Published diagnostic questionnaires for gambling disorder in German are either based on DSM-III criteria or focus on aspects other than life time prevalence. This study was designed to assess the usability of the DSM-IV criteria based Berlin Inventory of Gambling Behavior Screening tool in a clinical sample and adapt it to DSM-5 criteria.

**Methods:**

In a sample of 432 patients presenting for behavioral addiction assessment at the University Medical Center Mainz, we checked the screening tool’s results against clinical diagnosis and compared a subsample of *n*=300 clinically diagnosed gambling disorder patients with a comparison group of *n*=132.

**Results:**

The BIG-S produced a sensitivity of 99.7% and a specificity of 96.2%. The instrument’s unidimensionality and the diagnostic improvements of DSM-5 criteria were verified by exploratory and confirmatory factor analysis as well as receiver operating characteristic analysis.

**Conclusions:**

The BIG-S is a reliable and valid screening tool for gambling disorder and demonstrated its concise and comprehensible operationalization of current DSM-5 criteria in a clinical setting.

**Electronic supplementary material:**

The online version of this article (doi:10.1186/s12888-017-1349-4) contains supplementary material, which is available to authorized users.

## Background

Pathological gambling has been defined as a mental disorder by the American Psychiatric Association (APA) in the third edition of the Diagnostic and Statistical Manual of Mental Disorders (DSM-III) [1]. The classification and diagnostic criteria of pathological gambling have undergone revisions since then. In DSM-IV [2], pathological gambling criteria were revised and closely resembled those of substance dependence. However, it was still categorized as an impulse control disorder. Studies then showed that the elimination of one criterion (‘illegal activities to finance gambling’) and lowering the threshold for a diagnosis from 5 to 4 fulfilled criteria improved classification accuracy [3–5]. In DSM-5 [6], the name was changed to “gambling disorder”, the criterion referring to illegal activities omitted, the cut-off for diagnosis lowered to 4 fulfilled criteria, and it is now listed in the new category “Substance-Related and Addictive Disorders” (for a detailed review of the changing definitions and criteria, see [7]).

Diagnostic questionnaires for gambling disorder available in German include the South Oaks Gambling Screen (SOGS [8]; German version [9]), the short questionnaire for gambling behavior (“Kurzfragebogen zum Glücksspielverhalten”, KFG [10]), the Schwerin gambling questionnaire (“Schweriner Fragebogen zum Glücksspielen”, SFG [11]), and the Lie-Bet-Screen [12, 13]. Developed in the early eighties, the SOGS was the first validated screening instrument for the rapid screening for gambling disorder [8]. It operationalizes gambling problems by seven components based on DSM-III criteria: family and job disruption, lying about gambling wins and losses, default on debts, relying on others to relieve a desperate financial situation caused by gambling, borrowing from illegal sources, and committing an illegal act to finance gambling [14]. Its use quickly expanded to diverse settings and populations, including prevalence studies of gambling disorder in the general population [15–18]. As the most widely used screening instrument, it has been translated into many different languages [19–21]. Limitations of the SOGS, such as over-diagnosing gambling disorder relative to DSM-IV-based assessments, were discussed in detail by Strong et al. [14]. Like the SOGS, the KFG was developed in the early stages of gambling disorder research, adhering to the DSM-III criteria. Its items refer to present or past gambling behavior, detecting problematic gambling in currently abstinent respondents as well. The other instruments mentioned were designed for specific gambling-related issues: the SFG focuses solely on gambling behavior within the past seven days and is thus mainly used for detection of behavioral changes and treatment effectiveness in terms of pre-post-measurements. The Lie-Bet-scale consists of two items and was designed as the shortest possible screening tool.

The Berlin Inventory of Gambling Behavior (BIG, “Berliner Inventar zum Glücksspielverhalten”) was developed by the interdisciplinary addiction research group at the Charité – University Medical Center Berlin, based on the Questionnaire on Differentiated Assessment of Addiction (“Fragebogen zur Differenzierten Drogenanamnese”, FDDA), a self-rating instrument that provides the diagnosis of addiction as well as an overview of relevant information needed for the treatment of addiction [22]. The BIG includes gambling behavior questions and two diagnostic subscales - the BIG-PGS (10 item pathological gambling subscale, implementation of diagnostic criteria according to DSM-IV) and the BIG-GSS (according to ICD-10). Validated in an unpublished dissertation [23], Hesselbarth reported strong correlations for the BIG-PGS of *r* = .80 with the SOGS and *r* = .95 with the KFG and comparable proportions of normal, problematic and pathological gamblers. Its applicability to identify gambling disorder in a clinical sample has not been tested. The BIG-S is derived from the BIG-PGS and is meant to screen for gambling disorder. The screening items closely resemble the diagnostic criteria of the DSM-IV and aim for an economic, but more comprehensive operationalization than the BIG-PGS. Four of the DSM-IV gambling disorder criteria cover slightly differing aspects of the same behavior in one criterion (need to gamble with increasing amounts of time/increasing amounts of money, concealment/lying, jeopardizing a significant relationship/jeopardizing a job opportunity, different aspects of loss of control) and were thus converted into two items each, resulting in 14 instead of ten items overall. All items refer to the life time period, which enables the BIG-S to detect past gambling problems as well, asking whether the mentioned behavior or circumstance has ever been shown or observed at any time, in a dichotomous format (yes or no). For the four criteria operationalized by two items, affirmation of either one of the respective items (as well as affirmation of both) is interpreted as satisfying the criteria and scored as one point. The number of criteria met is summed up, resulting in scores between a minimum of zero and a maximum of ten, with a score of five or more indicating a gambling disorder (in accordance to the DSM-IV cut-off).

The purpose of this paper is to assess to the instrument’s usability in a clinical setting and its ability to identify gambling disorder in patients presenting for assessment. The probability of a patient to be classified in accordance with an expert’s diagnosis, especially for more extreme BIG-S scores, will be the main criterion to determine whether the BIG-S can be reliably used as a diagnostic tool. In order to validate the BIG-S as a diagnostic tool to identify gambling disorder, we assessed 432 patients presenting at our outpatient clinic for behavioral addictions (mainly gambling and internet-related disorders). Furthermore, the DSM-5 modifications to diagnostic criteria of gambling disorder will be tested with regard to diagnostic prediction accuracy, considering both the original DSM-IV version of the BIG-S (14 items, 10 criteria, cut-off 5 satisfied criteria) as well as a DSM-5 version (13 items, 9 criteria, cut-off 4 satisfied criteria).

## Methods

### Sample

All patients included presented for either gambling or internet-related disorders at the outpatient clinic for behavioral addiction ("Ambulanz für Spielsucht“), an outpatient treatment and research center at the Department of Psychosomatic Medicine of the University Medical Center Mainz. Patients were asked to fill out the BIG-S as part of the standard diagnostic questionnaires before the interview, demographics were collected as part of the basic documentation. All questionnaires were administered in German. In addition to the BIG-S, gambling and/or internet-related disorders and gambling/internet use history as well as comorbidities were assessed by a psychologist with extensive expertise in behavioral addiction during a 60 min diagnostic interview. Diagnoses were supervised by the head of the outpatient clinic for behavioral addiction and the director of the Department of Psychosomatic Medicine. The BIG-S score was computed and interpreted after the interview and separately from the medical diagnoses. The sample includes all gambling disorder patients from 2008 through 2014 with a clinical assessment of gambling disorder (*n* = 307, including *n* = 20 online gambling disorder). Of the 307 patients presenting for assessment of gambling disorder, *n* = 300 fulfilled DSM-IV criteria for gambling disorder in the interview, whereas *n* = 7 did not and were assigned to the comparison group. Beginning in 2014, internet-related disorder patients (*n* = 125) completed the BIG-S as well, forming the main part of the comparison group. Of those patients, *n* = 82 fulfilled criteria for internet-related disorder in the interview. None of the internet-related disorder patients fulfilled criteria for gambling disorder in the interview.

The “Gambling disorder” group (*n* = 300) averaged 33.32 years of age (SD 11.55). 89.3% were male, 22.1% of foreign nationality. 23.7% indicated completion of high school graduation, 1.4% were still in school. 46.0% were diagnosed with at least one more psychological disorder, the main comorbidities being mood disorders (30.3% of the Gambling disorder group), substance-related addictions (13.3%) and anxiety/stress related disorders (11.7%). The comparison group (*n* = 132) averaged 22.47 years of age (SD 7.79), with a comparable sex distribution (92.4% male) and a lower portion of foreign patients (5.8%). Completion of high school graduation was more common (36.6%), and 26.0% were still in school, corresponding with the younger age. Almost the exact same percentage was diagnosed with at least one more psychological disorder (46.2%), with two of the main comorbidities similar (23.5% mood disorders, 16.7% anxiety/stress related disorders, 1.5% substance-related addictions). 46.2% of the comparison group indicated that they had participated in some form of gambling before.

### Analyses

The factor structure of the 14 items was assessed by splitting the sample in half (randomly parallelizing the subsamples and matching distributions of treatment vs. comparison group, sex, age, nationality, and comorbidity rate) in order to perform exploratory and confirmatory factor analysis in different subsamples. Eigenvalues, scree plot, and factor loadings of a principal component analysis performed by SPSS with the first half of the sample were used to determine whether the 14 items (and resulting 10 gambling disorder criteria) were unidimensional. The factor structure was then evaluated by means of confirmatory factor analysis, testing the proposed model in AMOS with the second half of the sample.

#### Reliability

Cronbach’s alpha was calculated to evaluate the internal consistency of the 14 items, and the 10 resulting criteria used for the DSM-IV option, respectively.

#### Classification of gambling disorder patients using the BIG-S

Clinical assessment was replicated by the BIG-S according to DSM-IV (the 14 items operationalizing the 10 DSM-IV criteria were used, gambling disorder was indicated when a person scored 5 or more) and DSM-5 (the “Illegal Activities” item was removed, the resulting 13 items/9 criteria and a cut-off score of 4 were used).

#### Discriminant and convergent validity

The affirmation rate was calculated for each item for both the Gambling disorder and the comparison group. In order to assess how each item performs in discriminating between patients with and without gambling disorder, a phi correlation between item and group membership was computed for each item.

#### Classification consistency

The concordance of classification by clinical assessment and the BIG-S was evaluated by computing accuracy, sensitivity, specificity, and the Receiver-Operating Characteristic (ROC) curve for the BIG-S. Its accuracy is computed by dividing the sum of the true positives and true negatives by the total number of cases. Sensitivity is defined as true positives divided by the sum of true positives and false negatives. Specificity is defined as true negatives divided by the sum of true negatives and false positives. All statistical analyses were performed using SPSS and AMOS.

## Results

### Response to BIG-S items

**Fig. 1:**
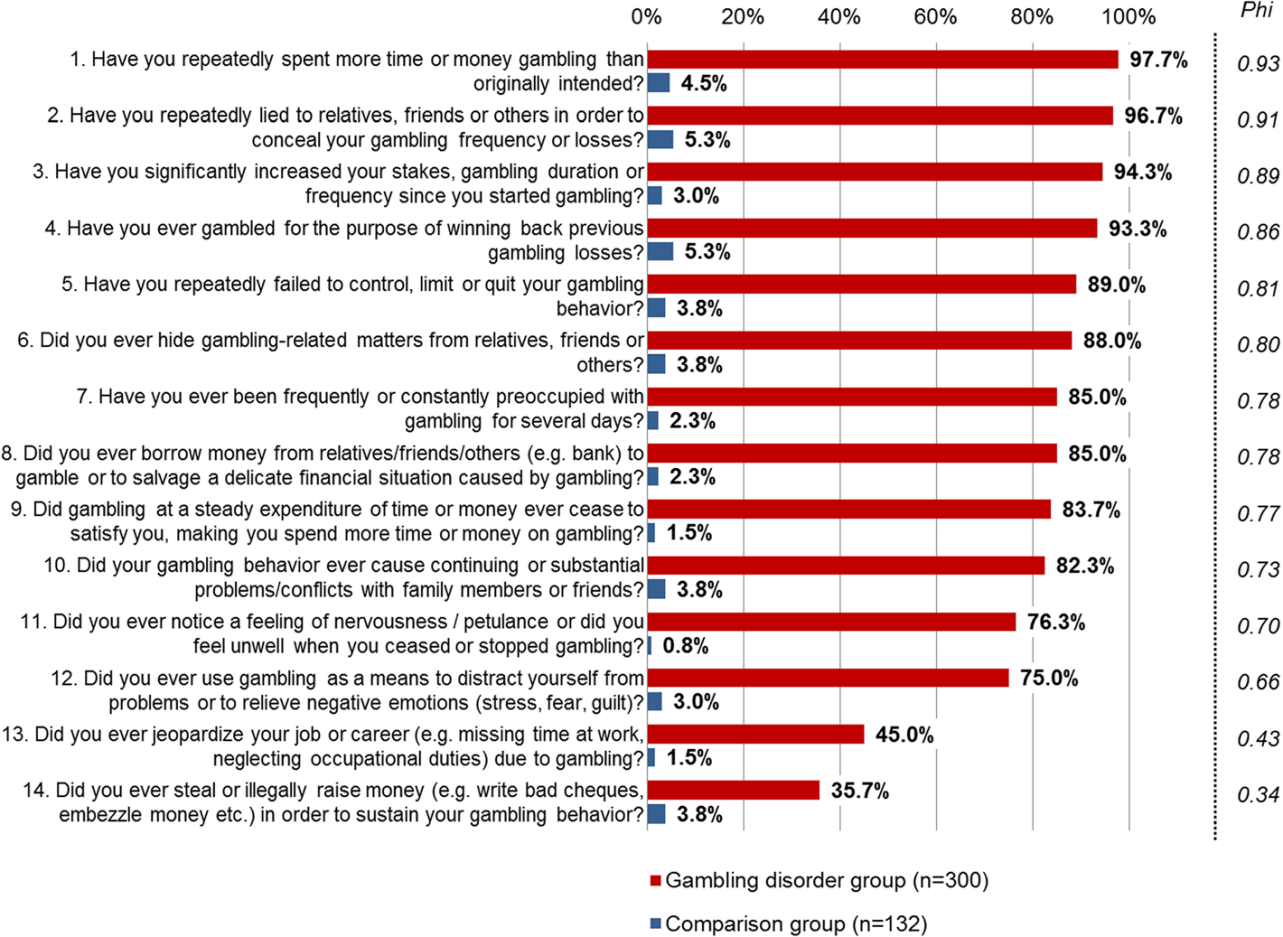
Affirmation rate of BIG-S items – Gambling disorder vs. comparison group

Figure 1 presents the items’ affirmation ratios for the Gambling disorder vs. comparison group.

Most of the BIG-S items were affirmed by the vast majority of the clinically diagnosed gambling disorder patients (see Fig. 1). Items related to loss of control (#1 and #5, 97.7% and 89.0%, respectively), concealment (#2 and #6, 96.7% and 88.0%, respectively), tolerance (#3 and #9, 94.3% and 83.7%, respectively), and chasing (#4, 93.3%) were most frequently endorsed. Only “jeopardized job/career” and “illegal activities” were affirmed by less than 50% of this group. No items stood out as prevalent in the comparison group (maximum affirmation 5.3%), but “withdrawal” appeared especially rare (#11, 0.8%). Phi was significant for all items, indicating their ability to discriminate between the Gambling disorder and comparison group. The lowest Phi scores were observed for “illegal activities” (#14, Phi = .34) and “jeopardized job/career” (#13, Phi = .43). All other items ranged between .66 and .93, with “spent more time/money than intended” leading all items (#1, Phi = .93). Other items with excellent discrimination values were “lying/concealing gambling behavior” (#2, Phi = .91) “significantly increasing gambling intensity” (#3, Phi = .89) and “chasing losses” (#4, Phi = .86).

### Factor structure

Principal component analysis with varimax rotation (Kaiser-Meyer-Olkin Measure of Sampling Adequacy = 0.95) resulted in a two factor solution according to the Eigenvalue criterion (factor 1: eigenvalue = 9.32, 66.6% of variance; factor 2: eigenvalue = 1.07, 7.6% of variance) for the 14 items. Factor loadings of 12 items on the first factor ranged from .73 to .92, while the two least frequently affirmed items (“jeopardized job/career”, “illegal activities”) loaded on the second factor (.80 and .86, respectively). The scree plot, however, clearly suggested a one factor solution. The 10 criteria resulting from the 14 items yielded a unidimensional scale with one factor according to the eigenvalue criterion and the scree plot (eigenvalue = 7.00, 70.0% of variance). The illegal activities-criterion had the lowest loading (.49), while all other criteria loadings ranged from .76 to .95.

**Fig. 2:**
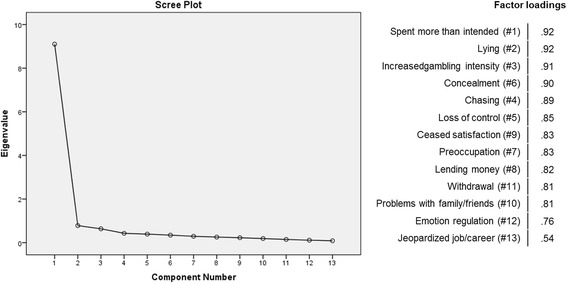
Factor analysis of the BIG-S items. Extraction Method: Principal Component Analysis, 13 items (item “illegal activities” removed), *n* = 216

Therefore this item was removed from the scale for a second factor analysis of the 13 remaining items (see Fig. 2). This yielded a unidimensional scale according to the eigenvalue criterion and the scree plot (eigenvalue = 9.10, 70.0% of variance). The “jeopardized job/career”-item had the lowest loading (.54), while all other item loadings ranged from .76 to .92.

Reliability was estimated using a measure of internal consistency, Cronbach’s alpha. Internal consistency for the 14 items was alpha = .96. The only possible improvements to this alpha would be omitting the items #14 (“illegal activities”) and #13 (“jeopardized job”), with marginal effects (+.004, and +.002 respectively). Internal consistency for the 10 criteria was alpha = 0.95. The only possible enhancement again proved to be omitting the item “illegal activities”, again with small effect (+.009).

Confirmatory factor analysis for the unidimensional model with 13 items without constraints revealed that incremental fit indices (CFI = .96, TLI = .96) were good, while absolute measures of fit indices were not satisfactory (RMSEA = .08, χ2 (65, *N* = 432) = 266.3, *p* < .001). Adding two constraints (*r* = .31 and *r* = .25 between errors of item #1and #4, and #5 and #12, respectively) improved all indices to a good model fit (CFI = .98, TLI = .97; RMSEA = .07, χ2 (63, *N* = 432) = 206.0, *p* < .001). Standardized factor loadings ranged from .24 (“jeopardized job”, item #13) and otherwise .48 to .86. All reported measures were superior to the model including the item “illegal activities”. The four items with the highest reported Phi also had the highest factor loadings in the exploratory (.89 to .92) and confirmatory factor analysis (.80 to .86, only items with loadings >.80).

Overall, statistical properties were slightly improved when adhering to DSM-5 criteria and omitting the “illegal activities” item. The scale’s unidimensionality and reliability were generally confirmed, especially for the DSM-5 version.

### Classification

**Fig. 3:**
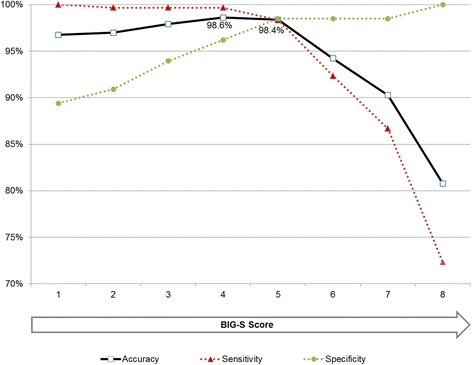
Accuracy, sensitivity and specificity of the BIG-S score (9 criteria)

When comparing the classification based on the BIG-S score and the clinical expert assessment after the interview, the best result was achieved by applying a cut-off according to DSM-5 specifications (9 criteria, score of 4 or more interpreted as gambling disorder): accuracy of 98.6% (0.2% false negatives, 1.2% false positives), a sensitivity of 99.7% and a specificity of 96.2% (compare Fig. 3). A cut-off of 5 for the 9 criteria option resulted in slightly lower accuracy (98.4%, 1.2% false negatives, 0.5% false positives), lower sensitivity (98.3%) and higher specificity (98.5%). The screener’s originally intended cut-off rule based on DSM-IV specifications (10 criteria, score of 5 or more interpreted as gambling disorder) yielded the same accuracy as the DSM-5 variant (98.6%), but with equal proportions of false negatives and false positives (0.7%, respectively), a sensitivity of 99.0% and a specificity of 97.7%.

None of the patients with a BIG-S score of 0 were diagnosed with gambling disorder after the interview. A BIG-S score of 5 or higher coincided with a gambling disorder diagnosis after the interview in 99.0% (DSM-IV) or 99.3% (DSM-5) of those cases.

**Fig. 4:**
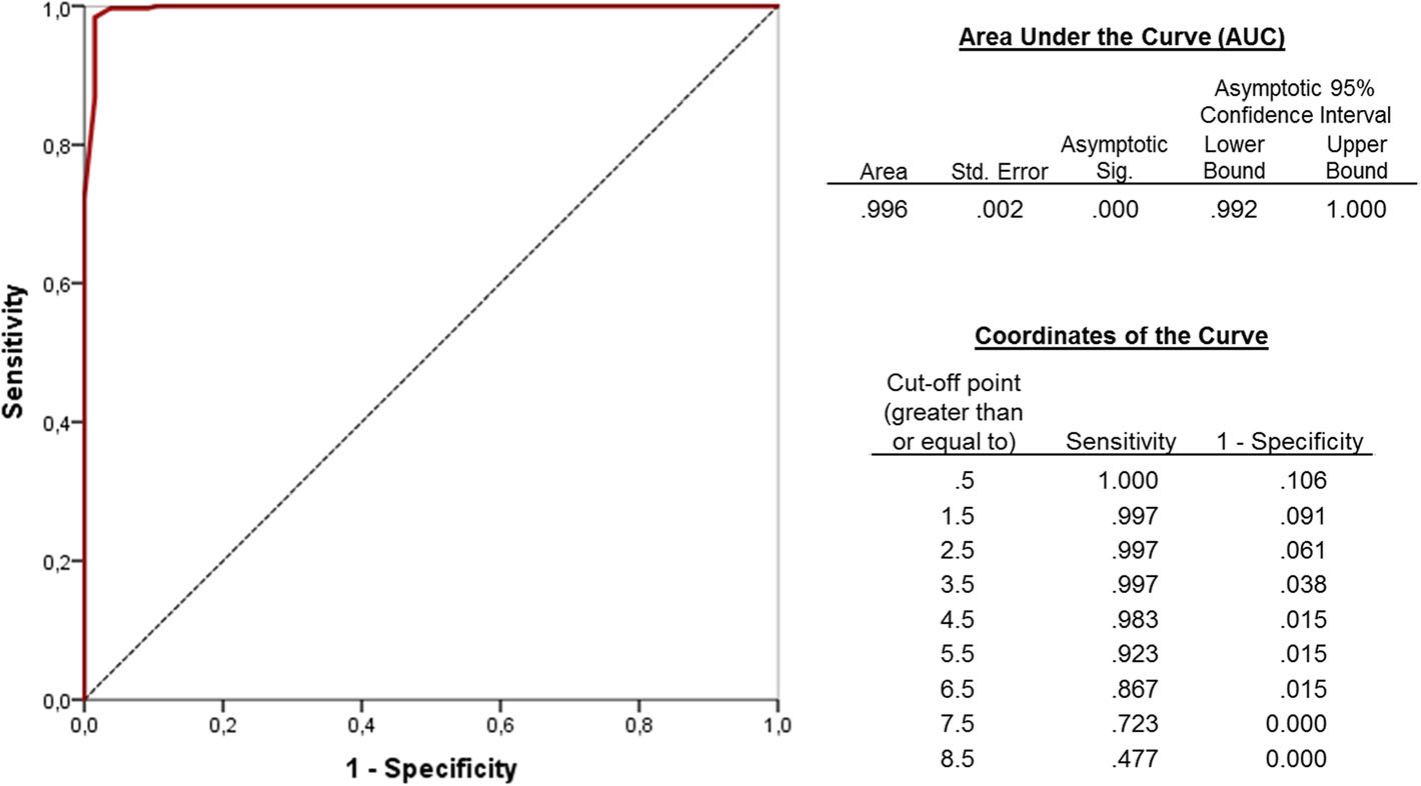
Receiver-Operating-Characteristic curve for DSM-5 specifications

The resulting Receiver-Operating-Characteristic (ROC) curves were virtually identical, with slightly better Area under the Curve (AUC) scores for the DSM-5 specifications (AUC = 0.996, 95% CI 0.992–1.000; see Fig. 4), with the DSM-IV specifications producing similar results (AUC = 0.994; 95% CI 0.986–1.000).

Overall, the BIG-S classification – whether used according to DSM-5 or DSM-IV specifications – showed very high accordance with the clinical assessment, which indicates the scale’s usability in the given context.

## Discussion and Conclusions

The present study aimed to validate the screening version of the Berlin Inventory of Gambling Behavior in a clinical sample of behavioral addiction patients. In conclusion, the instrument shows very good accuracy when compared to the clinical assessment as well as satisfactory reliability and validity, serving its purpose within the clinical context very well. Slight improvement of the instrument’s usability is accomplished when DSM-5 modifications are adopted, confirming previous research regarding DSM criteria for gambling disorder. As a considerable proportion of gambling disorder patients were immigrants, the BIG-S proves its comprehensible operationalization of DSM criteria and practicability as a diagnostic tool. The screening instrument is able to support clinical diagnosis by indicating no gambling disorder in the case of low scorers and alerting clinicians to very probable gambling disorder in high scorers.

The main modification in the DSM-5 definition for gambling disorder is the removal of the criterion „Has committed illegal acts such as forgery, fraud, theft or embezzlement to finance gambling“. The rationale for this change was the low prevalence of this behavior among individuals with gambling disorder, limiting its discriminatory power to the highest levels of gambling disorder severity [4]. Moreover, the threshold for a diagnosis was reduced from five to four criteria, resulting in a more accurate diagnosis of a gambling disorder [3, 5]. In line with these findings, better results in both EFA and CFA as well as scale reliability were achieved when the item “illegal activities” was excluded from the BIG-S. While some of the results point to the scale’s improvement when the item “endangering job opportunity” is removed, these improvements are deemed too insignificant to omit an item that represents one of two aspects of the DSM criterion “endangering important relationship/job opportunity”. The instrument demonstrated very good classification accuracy for all options examined. Improvement in false negative rate was obtained by adapting the scale’s computation to DSM-5 criteria and its cut-off point of 4, whereas classification accuracy remained the same (meaning that the improvement came at cost of false positive errors). False negative errors should be considered more severe in the diagnosis of gambling disorder (or any disorder in general), as they are likely to have greater and more serious consequences for the patient than false positive errors – particularly in a clinical setting, as the results of a self-report tool should always be validated in an interview when positive, but might be ignored when negative. Overall, the DSM-5 improvements were confirmed and the BIG-S should be used in the adapted version without the “illegal activities”-item and a cut-off of four points. Clinicians can be fairly confident that the respondent does not have a gambling disorder if his or her BIG-S score is 0, and can be fairly confident that the respondent does have a gambling disorder when his or her BIG-S score is 5 or higher.

Trivialization and dissimulation are common among behavioral addiction patients, e.g. the problematic aspects of the behavior are often mainly perceived by the patient’s relatives. This could be a reason for the observed “false negatives”, as some of the patients may have been motivated to remain undetected. Dissimulation is a lot more likely and easier to achieve in a “self evaluation”-questionnaire compared to a clinical interview.

Gambling and gaming translate to the same verb in German (“spielen”). The necessity to discriminate between the two corresponding behavioral addictions is based on the observation that internet use disorder patients related BIG-S items to their gaming behavior. E.g., one participant spent real money on in-game-purchases and thus may have interpreted his gaming as gambling, resulting in a “false positive”. That there were only very few false positives overall underscores the instrument’s usability in the specified setting.

A repeatedly identified result of gambling disorder research is the higher percentage of affected people within immigrant populations or citizens with migration background. In Germany, the representative PAGE study [24] found that prevalence among citizens with migration background was almost twice as high compared to the overall population - a finding replicated every two years by the BZgA monitoring [25–27], where the prevalence was found to be up to three times higher for citizens with migration background. As expected, foreign nationality was quite common within the Gambling disorder group. While at least a basic command of the German language is a necessity for personal assessment in the specified clinical setting, the high accuracy of the BIG-S seems to verify the instrument’s comprehensibility for patients of different linguistic levels.

### Limitations

We selected a comparison group based on the presence of behavioral addiction and because of online behavior variety within the internet-related disorder patients, creating a potential overlap between online and gambling behavior. However, there was mostly either excessive online gambling behavior or little gambling experience in this group, resulting in only few “borderline cases” (more than one but less than 4 items affirmed). The instrument’s specificity is lower when taking only control group patients with gambling experience into account (meaning if they indicated that they participated in some form of gambling before), but is still acceptable at 91,9%. Validating the BIG-S with a group of regular gamblers without - or with only slight - gambling problems would be interesting with regard to the instrument’s specificity. As there were only 10 respondents with BIG-S scores between 1 and 3 (with 9 correct negatives and 1 false negative), further research would help to ensure the positive tendency shown with regard to specificity in this “grey area” of gambling behavior. However, clinicians cannot draw definite conclusions for individuals with BIG-S scores of 4 solely based on the screener, because those had about a 50/50 chance of having a clinically diagnosed gambling disorder (7 cases with 3 false positives and 4 correct positives). Scores between 1 and 4 should always be checked within a clinical interview.

## Additional files

Additional file 1: Data file (raw data-set upon which the summary statistics in the manuscript are based; includes raw data and labels translated to English). (XLS 579 kb)

Additional file 2: BIG-S english translation (English language version of the developed instrument; includes scoring instruction). (DOCX 21 kb)

Additional file 3: Interview guideline (Guideline for the 60 min diagnostic interview upon which the psychologist’s assessment of gambling disorder and diagnoses are based). (DOCX 26 kb)
