# Supplementary material for: The Berlin Inventory of Gambling behavior – Screening (BIG-S): Validation using a clinical sample
Source: BMC Psychiatry. 2017 May 18;17:188. doi: 10.1186/s12888-017-1349-4 (PMC5437393; doi:10.1186/s12888-017-1349-4)
Supplement: Supplementary file 2 — BIG-S english translation (English language version of the developed instrument; includes scoring instruction). (DOCX 21 kb) [file 12888_2017_1349_MOESM2_ESM.docx]

*Instruction*: Please detail your gambling behavior by answering the following questions.

|  | | **yes** | | **no** | |
| --- | --- | --- | --- | --- | --- |
| 1. Have you significantly increased your stakes, gambling duration or frequency since you started gambling? | □ | | □ | |  |
| 2. Did gambling at a steady expenditure of time or money ever cease to satisfy you, making you spend more time or money on gambling? | □ | | □ | |  |
| 3. Have you repeatedly spent more time or money gambling than originally intended? | □ | | □ | |  |
| 4. Have you repeatedly failed to control, limit or quit your gambling behavior? | □ | | □ | |  |
| 5. Did you ever notice a feeling of nervousness / petulance or did you feel unwell when you ceased or stopped gambling? | □ | | □ | |  |
| 6. Have you ever been frequently or constantly preoccupied with gambling for several days? | □ | | □ | |  |
| 7. Have you ever gambled for the purpose of winning back previous gambling losses? | □ | | □ | |  |
| 8. Did you ever use gambling as a means to distract yourself from problems or to relieve negative emotions (stress, fear, guilt)? | □ | | □ | |  |
| 9. Did you ever jeopardize your job or career (e.g. missing time at work, neglecting occupational duties) due to gambling? | □ | | □ | |  |
| 10. Did your gambling behavior ever cause continuing or substantial problems/conflicts with family members or friends? | □ | | □ | |  |
| 11. Have you repeatedly lied to relatives, friends or others in order to conceal your gambling frequency or losses? | □ | | □ | |  |
| 12. Did you ever hide gambling-related matters from relatives, friends or others? | □ | | □ | |  |
| 13. Did you ever borrow money from relatives/friends/others (e.g. bank) to gamble or to salvage a delicate financial situation caused by gambling? | □ | | □ | |  |
| 14. Did you ever steal or illegally raise money (e.g. write bad cheques, embezzle money etc.) in order to sustain your gambling behavior? | □ | | □ | |  |
