# Supplementary material for: The Berlin Inventory of Gambling behavior – Screening (BIG-S): Validation using a clinical sample
Source: BMC Psychiatry. 2017 May 18;17:188. doi: 10.1186/s12888-017-1349-4 (PMC5437393; doi:10.1186/s12888-017-1349-4)
Supplement: Supplementary file 3 — Interview guideline (Guideline for the 60 min diagnostic interview upon which the psychologist’s assessment of gambling disorder and diagnoses are based). (DOCX 26 kb) [file 12888_2017_1349_MOESM3_ESM.docx]

**History** Name: Age: Date:

1. Medical report to patient / referring doctor:
2. Current symptoms (cause of appointment/“sent“?, main gam(bl)ing form?, How long ago?, financial situation?) and history of gam(bl)ing disorder (onset, progression, symptom-free phases, quit attempts, comorbidity)

Tolerance

Loss of control

Chasing

Emotion regulation

Borrowing money

concealment

jeopardizing. relation/job

preoccupied

withdrawal

(Illegal activities)

1. Treatment goal / expectation?
2. Pre-treatment: psychotherapy (currently – past), other
3. Physical disorders (current / past):
4. Medication (current / past)
5. Drug usage (incl. alcohol, tobacco)
6. Suicidal tendency

current:

past:

1. Partnership (since when, quality/problems, partner aware of gam(bl)ing behavior?)
2. Children (age / quality of relationship)
3. Family of origin (country of origin, parents and siblings: quality of relationship, aware of gam(bl)ing behavior?)
4. Occupation / Education (Progress / Problems)
5. Financial Situation / Debts
6. Activities / Social life / Sport
7. Other symptoms, comorbidities:

**🡪 Recommendation 🡪adopted by patient**

- Monitoring of gam(bl)ing behavior / re-assessment if necessary O
- psychosocial counseling O
- Waitlist group therapy (call for next group) O
- Initiate application for group therapy O
- inpatient rehabilitation (gam(bl)ing specific) O
- inpatient rehabilitation (psychosomatic, inhouse) O
- Caritas / Debt counseling O
- Outpatient psychotherapy (practicing psychotherapist) O
- Psychiatrist O
- Other: O

**🡪 Follow-up visit / call**: no

yes date:

**🡪 Diagnoses:**
